# Antibacterial activities of oregano essential oils and their active components

**DOI:** 10.3389/fphar.2025.1579283

**Published:** 2025-04-08

**Authors:** Lei Tao, Yan Liang, Zhi Xia, Xinsheng Wang, Xiaodong Wang, Zhe Chao, Jie Guo

**Affiliations:** ^1^ Lanzhou Vocational Technical College, School of Health, Lanzhou, China; ^2^ College of Agronomy, Henan Agricultural University, Zhengzhou, China; ^3^ Oregano Technology Research Institute, Henan Shennong Authentic Medicinal Materials Co., Ltd, Zhengzhou, China; ^4^ College of Veterinary Medicine, Gansu Agricultural University, Lanzhou, China

**Keywords:** oregano essential oils, carvacrol, thymol, antibacterial activity, synergistic effect

## Abstract

**Background:** Oregano essential oils (OEOs) and their biological active components are of great interest due to their potent pharmaceutical and antibacterial activities.

**Methods:** OEOs were extracted from wild and cultivated oregano with white or purple flower using our extraction process. We investigated the *in vitro* antibacterial effects of OEOs and the main active components, carvacrol and thymol. The synergistic effects of carvacrol and thymol were evaluated using checkerboard assay, time-kill assays and systemic infection mice model. The synergistic mechanism was also revealed using scanning electron microscopy (SEM) assay and DAPI/PI staining.

**Results:** Essential oil extracted from wild and cultivated oregano with white flower exhibited potent antibacterial activities against standard strains of Gram-positive and -negative bacteria, with minimum inhibitory concentrations (MICs) range from 0.25-1 mg/mL. The antibacterial activities of carvacrol were obvious higher than that of thymol with MICs values of 0.005-0.04 mg/mL. Carvacrol combining with tobramycin exhibited highly promising synergistic effects (with FICI = 0.25 against *E.coli* and 0.125 against MRSA) which were further confirmed by the time-kill assays. In the systemic infection mice model, carvacrol combining with tobramycin exhibited potent *in vivo* antibacterial effects, with significantly improving the survival rate of mice, reducing the MRSA load and alleviating the pathological changes in the lungs of the infected mice. Preliminary explorations for synergistic mechanism suggested that the enhanced antibacterial potential of tobramycin might be attributed to carvacrol with the ability to perforate membrane and induce holes on it.

## 1 Introduction

The increasing development of multidrug-resistant (MDR) strains has become a serious and growing threat to human health (World Health Organization, 2022; Xia et al., 2023), especially the notorious “ESKAPE” pathogens, including *Escherichia coli* (*E*. *coli*), *Staphylococcus aureus* (*S*. *aureus*), *K. pneumoniae* (*Klebsiella pneumoniae*), *A. baumannii* (*Acinetobacter baumannii*), *P. aeruginosa* (*Pseudomonas aeruginosa*) and *Enterococcus faecium* (*E*. *faecium*) (Gallardo-Macias et al., 2024; Rossolini et al., 2014). Moreover, most antibiotics are losing their efficiency because of the wide-spreading bacterial resistance (Astolfi et al., 2017). Therefore, it is imperative to develop effective antimicrobial agents with low probability to induce bacteria to develop resistance (Lewis, 2020; Zhang et al., 2023).

Compared to synthetic antimicrobial agents in recent years, natural products have been recognized to be most promising choice for antimicrobial agents and extensively studied due to their low toxicity and broad spectrum of action (Chen et al., 2024). *Origanum vulgare* L. (commonly known as oregano) is a perennial plant belonging to the Lamiaceae family and widespread throughout Asia, particularly in Iran (Saffarian et al., 2024). Literature data have revealed that this plant exhibits several useful therapeutic properties (Morshedloo et al., 2018; Abdulmajeed, 2015). Oregano essential oils (OEOs) are extracted from its leaves by steam distillation. Significant research has proved that OEOs displayed many useful biological activities associated with its chemical composition, including antibacterial activities, antioxidant, anti-inflammatory, antifungal, antiviral and antihyperglycemic (Saffarian, et al., 2024; Bautista-Hernandez et al., 2021; Marin-Tinoco et al., 2021).

The remarkable antibacterial properties of OEOs are particularly important and have been extensively studied in light of the emergence of MDR strains associated with infections (Marin-Tinoco et al., 2023; Sirati et al., 2024). Because of high content of phenolic derivatives, OEOs shows a wide spectrum of antimicrobial activity (Nagmetova, et al., 2020). Studies have corroborated the efficacy of OEOs against various foodborne pathogens, including *Salmonella*, *Listeria monocytogenes* and *E*. *coli* (Saffarian et al., 2024; Leyva-López et al., 2017). The inhibitive effects of OEOs against pathogens make it promise to extend spoilage of meat products into which incorporate oregano extracts or OEOs (Stojanovic et al., 2024; Ozogul et al., 2020). Adding OEOs into toothpaste could completely disrupt biofilms produced by cariogenic *Streptococcus mutans* (*S. mutans*) (Oliveira et al., 2020; Yuan et al., 2023).

**FIGURE 1:**
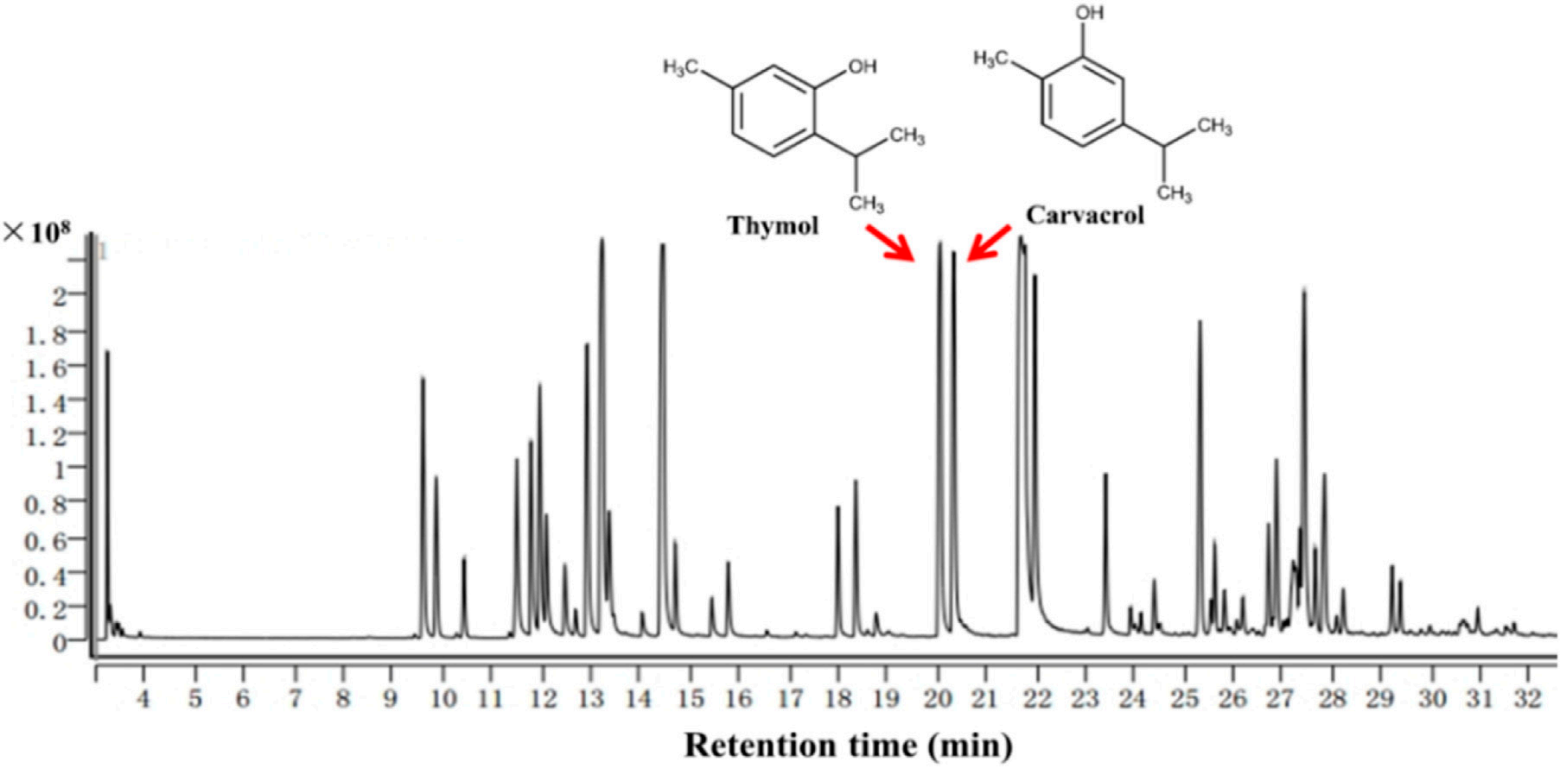
Gas chromatogram of OEO.

Carvacrol and thymol (Figure 1), the promising components of OEOs, are monoterpenoid compounds with a single phenolic ring formed from the bonding of two isoprene molecules (Peixoto-Neves et al., 2009; Zheng et al., 2024). They display significantly inhibit the growth of both Gram-positive and -negative bacteria, as well as viruses and fungi (Cid-Perez et al., 2024). The synergism of carvacrol or thymol with antibiotics is a useful property to control antibiotic resistance or lowering the dosage or toxicity of certain antibiotics (Kachura and Suntres, 2020). The frequently reported mechanism of antibacterial action is the disruption of cell wall and membrane that leads to the leakage of intracellular contents and bacteria lysis. Inhibition of efflux pumps is another proposed antibacterial mechanism, by which results in the disturbances in ATP balance (Helander et al., 1998; Walsh et al., 2003; Kachura and Suntres, 2020).

However, the concentrations of carvacrol and thymol in OEOs have some discrepancies when different extraction processes or producing areas of oregano are used, which may result in diversity in the antibacterial activity of OEOs (Sakkas and Papadopoulou, 2017; Mora-Zúñiga et al., 2022). In this regard, the aims of this work were to investigate the *in vitro* antibacterial effects of OEOs extracted from wild and cultivated oregano with different colors of flowers using our extraction process. Furthermore, considering the antibacterial mechanisms, the synergistic effects of carvacrol/thymol and common antibiotics were further evaluated in *vitro* and in a murine infection model.

## 2 Materials and methods

### 2.1 Plant material and the preparation of essential oils

Wild and cultivative *O. vulgare* L. grow in Yexian County of Henan province, China, located at latitude: 33.62^o^ N, longitude: 113.35^o^ E. These plants were all collected in a single day in the September. In this study, cultivative *O. vulgare* L. were divided into white flower and purple flower, and extracted separately. Oregano essential oil was obtained by hydrodistillation process using Clevenger type apparatus for 4 h. The obtained oils were further isolated using a separatory funnel and dried with anhydrous sodium sulfate for further analysis.

### 2.2 Determination of thymol and carvacrol in oils

The determination of chemical composition content in oils and chromatographic separation was performed on Agilent 8,890–7250 GC-QTOF (Agilent Technologies, Santa Clara, CA). An HP- 5MS UI (15 m × 0.25 mm × 0.25 μm) was employed and the column temperature was maintained at 180°C. Sample (1 µL) was injected at a split mode of 20:1 and helium was used as gas carrier at 1.0 mL/min. Multi-mode injection (MMI) temperature was set to 300°C, and the temperature programming was as follows: the temperature remained at 45°C for 1 min, linearly increasing to 300°C at 10 ^o^C/min rates and holding for 10 min. Electron ionization was 70 eV, with a mass range of 40–500 amu. Ion source and quadrupole temperature were set to 230^o^C and 150^o^C, respectively. The identification of the compounds was carried out by comparing their mass spectra with those of the NIST-MS and WILEY-MS library, considering a quality coincidence >85% (Özogul et al., 2022).

### 2.3 Bacterial strains

Standard strains of Gram-positive and negative bacteria were used in this study, including *S. aureus* ATCC 25923 (*S*. *aureus*), methicillin-resistant *S. aureus* BNCC 337371 (MRSA), methicillin-resistant *Staphylococcus epidermidis* ATCC 51625 (MRSE), *Enterococcus faecalis* ATCC*51299* (*E*. *faecalis*), *E. faecium* ATCC 35667 (*E*. *faecium*), *Streptococcus agalactiae* ATCC 27956 (*S*. *agalactiae*), *E. coli* ATCC 25922 (*E*. *coli*) and *Salmonella typhimurium* SL1344 (*S*. *typhimurium*). The clinical isolates of MRSA (56 strains) were isolated from dairy farms in four different provinces in China. These strains were routinely cultured in Mueller Hinton broth (MHB) and agar or Blood Agar.

### 2.4 Animals

Seventy-five specific pathogen-free Balb/c mice weighing 18–20 g (six-week-old) were purchased from the Laboratory Animal Center of Lanzhou University, and the animal studies were carried out in accordance with the ethical principles of animal research and approved by the Ethics Committee of Laboratory Animal Center of Lanzhou University (No. SCXK 2024-0007). Animals were kept in clean, stainless steel cages with free access to food and water under 23°C conditions, with a constant 12 h light-dark cycle. Animals were used for the experiments after being acclimatized for 7 days in compliance with the ARRIVE guidelines (Percie du Sert et al., 2020).

### 2.5 Minimum inhibitory concentration (MIC) determination

The MICs of essential oils extracted from wild oregano (OEO-1), cultivated oregano with white flower (OEO-2) and cultivated oregano with purple flower (OEO-3), the commercial OEO (OEO-4, purchased from Aromaaz International Private Limited, New Delhi, India), as well as carvacrol and thymol, were determined by micro-dilution technique according to the Clinical and Laboratory Standards Institute (CLSI) guidelines (Clinical Laboratory Standards Institute, 2012). Briefly, the above mentioned oil and compounds were dissolved in 20% DMSO to a concentration of 128 mg/mL, respectively. Subsequently, 100 μL solutions were diluted twofold with 100 μL MHB to provide 11 serial dilutions. The logarithmic phases of strains, including standard strain and 56 strains of clinical MRSA were diluted to 10^5^–10^6^ CFU/mL in MHB. Then, 100 μL volumes of the tested strains were added to the serial dilutions. The mixtures were then incubated at 37°C for 18–24 h. The MIC was determined by optical density (OD) measurements at a wavelength of 492 nm as the lowest concentration that resulted in no bacterial growth. The same procedure was repeated three times.

### 2.6 Checkerboard assay

After determining the MICs of selected antibiotics (tobramycin, chloramphenicol, ampicillin, polymyxin, tiamulin, vancomycin and cefepime) against MRSA and *E. coli* using broth dilution method, the synergistic effects of carvacrol/thymol with these antibiotics were determined by the checkerboard method as described in previous works (Zhang et al., 2022; Zhang et al., 2024). The strains were cultured and diluted with fresh MH medium to 1 × 10^6^ CFU/mL. The diluted bacterial suspension (100 µL) was mixed with 50 µL thymol/carvacrol and 50 µL antibiotics with two-fold serially diluted concentrations using 96-well plate. The plate was then sealed and placed in a constant temperature incubator at 37°C for 20 h. The single drug MIC and the MIC values of the best combination effect (MICA combined with MICB) were recorded. The fractional inhibitory concentration index (FICI) was calculated by the following equation: FICI = FICA + FICB, in which FICA = MIC of carvacrol or thymol in combination/MIC of thymol or carvacrol alone; FIC B = MIC of antibiotic in combination/MIC antibiotic alone. The synergistic effects of thymol/carvacrol was categorized as synergistic when the FICI was ≤0.5, additive when 0.5 < FICI ≤1, indifferent when 1 < FICI ≤4, and antagonistic when FICI >4 (Leber, 2016).

### 2.7 Bactericidal time-kill kinetics

*Escherichia coli* ATCC 25922 and MRSA BNCC 337371 were cultured in MHB at 37°C for 8 h with shaking and diluted to approximately 1 × 10^6^ CFU/mL. Test compounds (with the final concentrations of 1 × MIC and 6 × MIC) were inoculated with the aliquots of bacteria resuspended in fresh media. After specified time intervals (0, 2, 4, 6, 8, 12 and 24 h), cultures incubated at 37°C with 200 rpm shaking were serially diluted by 10-fold in 0.9% saline. The resulted serial dilutions (10 μL) were plated on sterile MH agar plates and incubated at 37 °C for 24 h. The viable colonies were counted and represented as log_10_ (CFU/mL). The same procedure was repeated in triplicate on different days.

### 2.8 Scanning electron microscopy (SEM) observations

MRSA BNCC 337371 were incubated in the MH broth medium at 37°C, with a shaking speed of 200 rpm for 16 h. Carvacrol, tobramycin, and carvacrol combining with tobramycin (4 × MIC) were added to culture for 4 h. The precipitate was collected by centrifuging at 8,000 rpm for 5 min. The obtained sample was then washed twice with PBS. After centrifuging at 8,000 rpm for 5 min, bacteria were fixed using 2.5% pentanediol. The cells were subsequently washed with 0.1 M PBS and dehydrated with 30%, 50%, 70%, 80%, 90%, 95% and 100% ethanol. The bacterial morphology was finally analyzed using SEM.

### 2.9 DAPI/PI staining

The fluorescent dyes 4′,6-diamidino-2-phenylindole (DAPI) and propidium iodide (PI) were used to further explore the effect of compounds on cell membrane permeability. Carvacrol, tobramycin, and carvacrol combining with tobramycin (4 × MIC) were added to MRSA (BNCC 337371) suspension, and negative control only used PBS (pH = 7.2–7.4) without any drug. After incubation at 37°C and centrifuging at 200 rpm for 1 h, DAPI (10 μg/mL) and PI (20 μg/mL) were mixed in suspension in the dark, and then the suspension was cultured at 0°C for 20 min. Finally, the results were recorded on a fluorescence microscopy.

### 2.10 Murine systemic infection model

The SPF Balb/c mice were randomly divided into five groups and rendered neutropenic upon treatment with 150 mg/kg cyclophosphamide intraperitoneally for 4 days and with 100 mg/kg for 1 day prior to inoculation, respectively. The neutropenic mice were then intraperitoneally injected with a 0.5 mL MRSA inoculum containing 1 × 10^8^ CFU/mL. After 1 h of infection, the mice in four groups were intravenously administered tobramycin dissolved in saline and carvacrol mixed with tobramycin (weight ratio = 10:1) which was dissolved in 0.5 mL of vehicle (comprising DMSO:Tween-80:sterile water in a ratio of 5:5:90) at doses of 30 and 10 mg/kg body weight, respectively. Mice in the rest group were used as control and only intravenously administered with the same volume of vehicle. For the survival rate experiment, animals (n = 10) were monitored twice daily for symptoms and mortality. Survival at 7 days after infection was used as the endpoint. To determine MRSA in tissues and histological analyses, treated mice (n = 5) at 3 days after infection were anesthetized with excess CO_2_ and euthanized *via* exsanguination. Organ samples (kidneys, liver and lungs) from surviving mice and those that died during the treatment were prepared for the tissue staining experiment.

### 2.11 Statistical analysis

Statistical analysis was conducted using IBM SPSS Statistics for Windows version 24.0 (SPSS Inc., Chicago, USA). One-way analysis of variance was utilized to analyze the data, followed by Dunnett’s posthoc tests when applicable (IBM SPSS, 2012). A *P*-value of <0.05 was considered statistically significant, and a *P*-value of <0.01 was considered highly significant.

## 3 Results

### 3.1 Yield of essential oils and the content of carvacrol and thymol in oils

After extraction from wild and cultivated *O. vulgare* L., essential oils were obtained varying from 3.58% to 4.12% yielding with no significantly difference. Dozens of chemical compounds that represented over 99% of its total composition were identified *via* GC-MS (Figure 1). Monoterpenes and sesquiterpenes were proved to be the most abundant bioactive components, in which thymol and carvacrol comprised 16.75% and 21.98% content, respectively.

### 3.2 MIC of OEOs, carvacrol and thymol

Medicinal plants have been recognized as the major sources for the prevention and treatment of infectious diseases. To confirm the essential oils harboring antimicrobial activities that were extracted from cultivated oregano and wild oregano, we determined their MICs against six Gram-positive and two Gram-negative strains. The MICs results are presented in Table 1. All the tested OEOs exhibited moderate antibacterial efficacy against Gram-positive and negative strains, with MIC range from 0.25–8 mg/mL. Essential oils extracted from oregano with white flower, whether it came from cultivated or wild, displayed the similar MICs (0.5–1 mg/mL). To our surprise, except against MRSA and *S. agalactiae*, the MICs of essential oil that extracted from purple flower oregano (OEO-3), with MICs range from 2–8 mg/mL, showed markedly lower antibacterial activities than that of essential oil extracted from white flower oregano (OEO-1 and OEO-2). In general, the commercial oil (OEO-4) possessed the higher antibacterial activities than the other oils. The antibacterial activities of carvacrol against the test strains were apparently higher than that of thymol, especially against *S. aureus*, *E. faecium* and *S. agalactiae*, with MIC values of ≤0.04 mg/mL.

**TABLE 1:** MICs of OEOs, carvacrol and thymol.

Oils or compounds	MICs (mg/mL)							
	*S. aureus*	MRSA	MRSE	*E. faecalis*	*E. faecium*	*S. agalactiae*	*E. coli*	*S. typhimurium*
OEO-1	0.5	1	1	1	0.5	0.25	1	1
OEO-2	0.5	1	0.5	1	0.5	0.25	1	1
OEO-3	4	0.5	2	4	8	0.5	8	8
OEO-4	0.25	0.25	0.25	0.5	0.25	0.25	0.5	0.25
Thymol	0.04	0.16	0.04	0.08	0.16	0.08	0.16	0.08
Carvacrol	0.005	0.01	0.02	0.04	0.005	0.005	0.01	0.01

**FIGURE 2:**
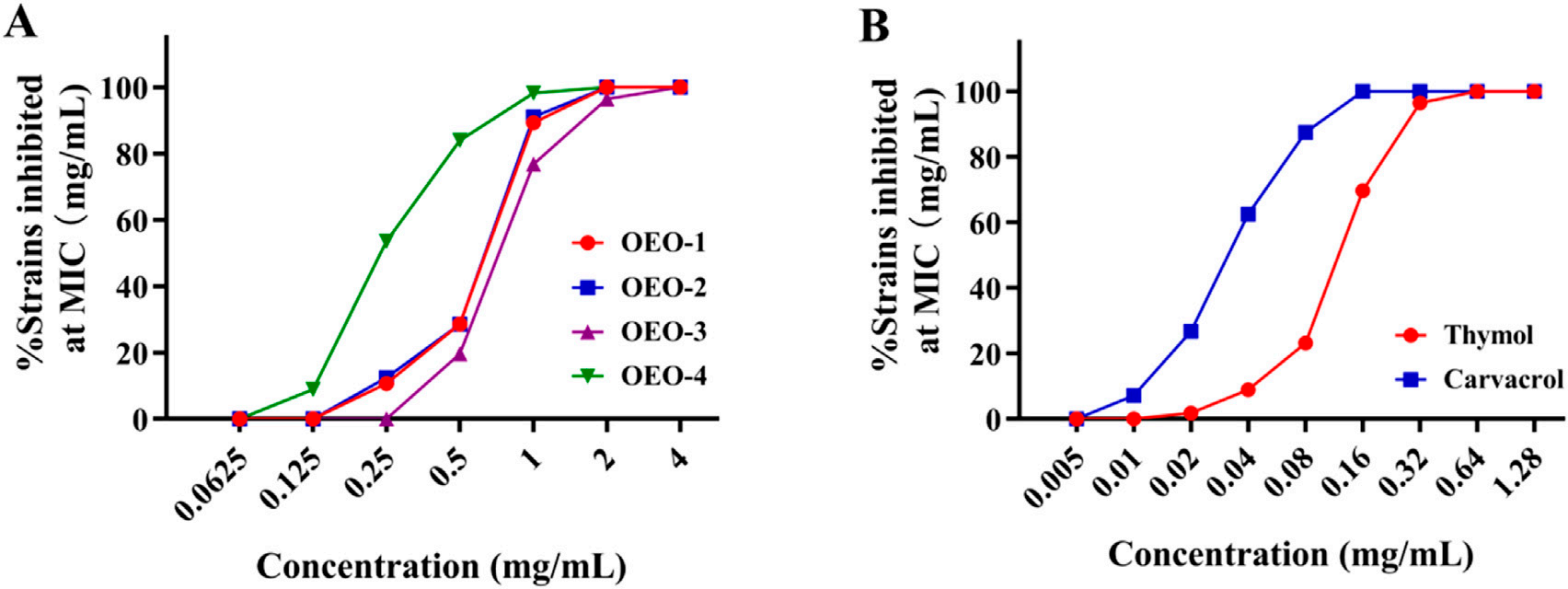
Inhibition rate of OEOs **(A)** and their bioactive components **(B)** against clinical isolates of MRSA.

For further validating the antibacterial activities of OEOs, we continued to test MIC against the 56 clinical isolates of MRSA. The inhibition rate of OEOs, carvacrol and thymol against these isolates that reflected their antibacterial activities were showed in Figure 2, and the MIC data were showed in Supplementary Table S1. The results revealed that the antibacterial activities of four essential oils or the two bioactive components against the clinical isolates agreed well with that against the standard MRSA. OEO-1 and OEO-2 showed the similar activities against all these strains with MIC with 0.25–2 mg/mL, slightly higher than that of OEO-3 (MICs were 0.5–4 μg/mL) but much lower than that of OEO-4 (MICs were 0.125–1 μg/mL). At the 0.5 μg/mL, OEO-4 inhibited 83.93% strains, while OEO-1 and OEO-2 inhibited 28.57% strains. However, OEO-3 showed the lowest antibacterial activity with only 19.64% strains inhibited at this concentration (Figure 2A). The MICs values of carvacrol and thymol were 0.01–0.16 mg/mL and 0.02–0.64 μg/mL, respectively. Carvacrol displayed higher antibacterial activities than that of thymol within the collections of clinical isolates (Figure 2B).

### 3.3 Synergistic effects of carvacrol and thymol with antibiotics

**FIGURE 3:**
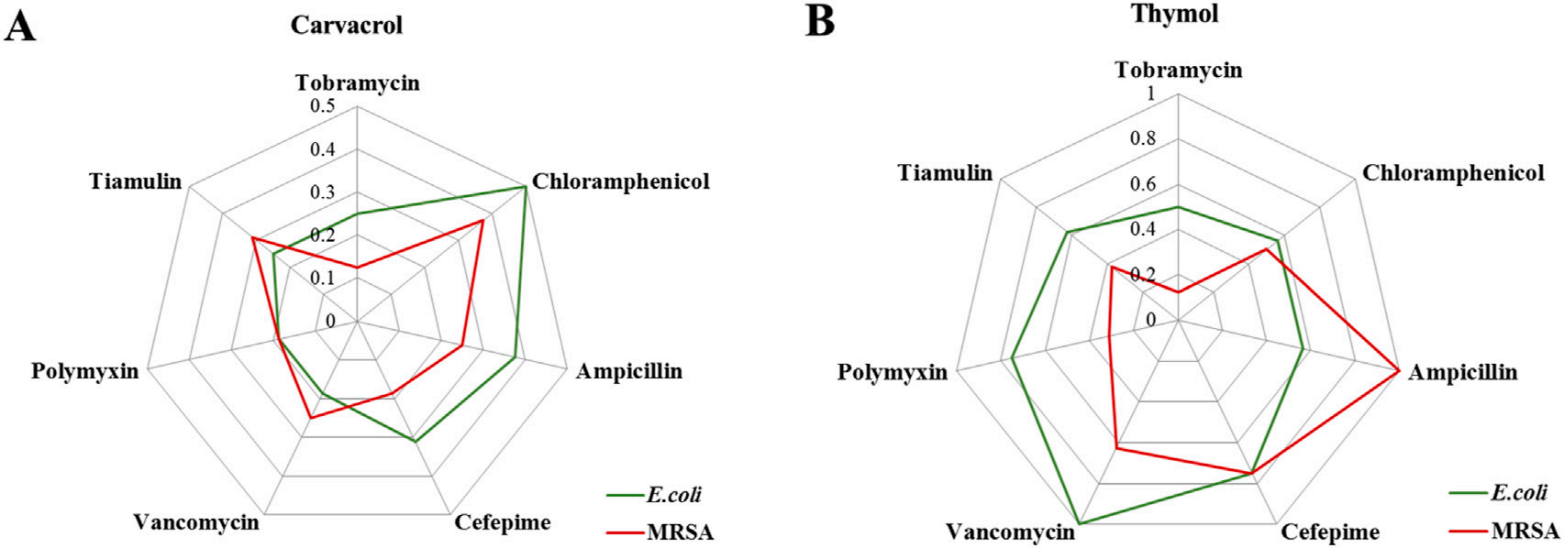
Radar chart of FICI values which revealed the synergistic effects of carvacrol **(A)** and thymol **(B)** with selected antibiotics against *E. coli* and MRSA, respectively.

Given the potent *in vitro* antibacterial activity of carvacrol and thymol, we subsequently used checkerboard method to evaluate their synergistic effects and seven classes of common antibiotics, including tobramycin (aminoglycoside antibiotics), chloramphenicol (chloramphenicol antibiotics), ampicillin (β-lactam antibiotic), cefepime (cephalosporin antibiotic), vancomycin (glycopeptide antibiotics), polymyxin (polypeptide antibiotics) and tiamulin (pleuromutilins), against *E. coli* and MRSA, respectively. As shown in Figure 3 and Supplementary Figure S1, carvacrol displayed significantly synergistic effects for the selected seven antibiotics, with FICI values from 0.1875 to 0.5 for *E. coli* and from 0.1875 to 0.375 for MRSA, respectively. Notably, carvacrol dramatically enhanced the antimicrobial activity of tobramycin (FICI = 0.25 against *E. coli* and 0.125 against MRSA), polymyxin (FICI = 0.1875 against *E. coli* and MRSA) and vancomycin (FICI = 0.1875 against *E. coli* and 0.25 against MRSA). However, thymol displayed reduced synergistic effects for antibiotics, with FICI values from 0.5 to 1 for *E. coli* and from 0.125 to 1 for MRSA, respectively. These results revealed the strong potential of carvacrol for increasing the susceptibility of *E. coli* and MRSA to antibiotics as an antimicrobial adjuvant.

### 3.4 Time-kill analysis

**FIGURE 4:**
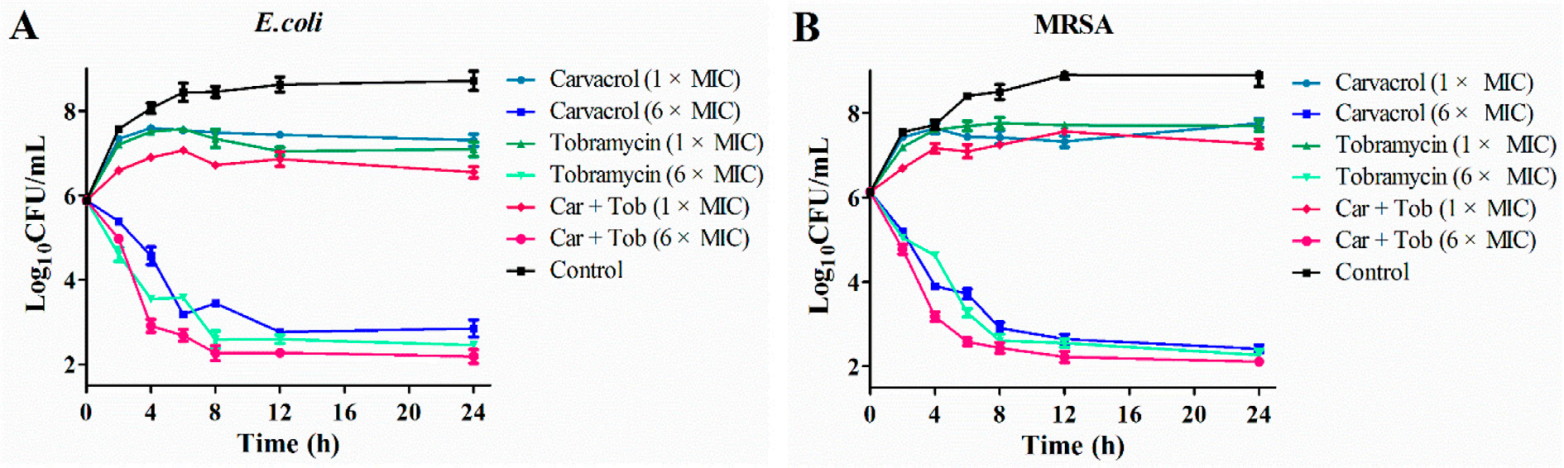
Time-kill analysis of carvacrol, tobramycin and carvacrol combining with tobramycin (Car + Tob) against *E. coli* **(A)** and MRSA **(B)**.

Based on the results from synergistic effects study, we chose 1.25 μg/mL carvacrol and 0.125 μg/mL tobramycin as the best synergistic combinations which displayed the ideal antibacterial activity against *E. coli and* MRSA, and used to further evaluate. To reveal the synergistic effect, we compared the bacterial killing kinetics of carvacrol, tobramycin and carvacrol combining with tobramycin. The strains were treated with compounds at the concentration of 1 × and 6 × MIC to count the number of bacteria over 24 h (Figure 4). The results showed that1 × MIC of carvacrol, tobramycin, and carvacrol combining with tobramycin slowed bacterial propagation when compared to the control. At the concentration of 6 × MIC, all the tested compounds showed relatively bacteriostatic kinetics against *E. coli* (Figure 4A) and MRSA (Figure 4B). Carvacrol combining with tobramycin showed the highest antibacterial activities than carvacrol and tobramycin alone, obtaining a 3.62 log reduction in the bacterial counts of *E. coli* and a 3.70 log reduction in the bacterial counts of MRSA within 8 h. These findings demonstrated that carvacrol combining with tobramycin could quickly kill bacteria *via* time dependent and can be used to efficiently treat the infections of troublesome nosocomial pathogens, such as *E. coli* and MRSA.

### 3.5 Antibacterial mechanism

**FIGURE 5:**
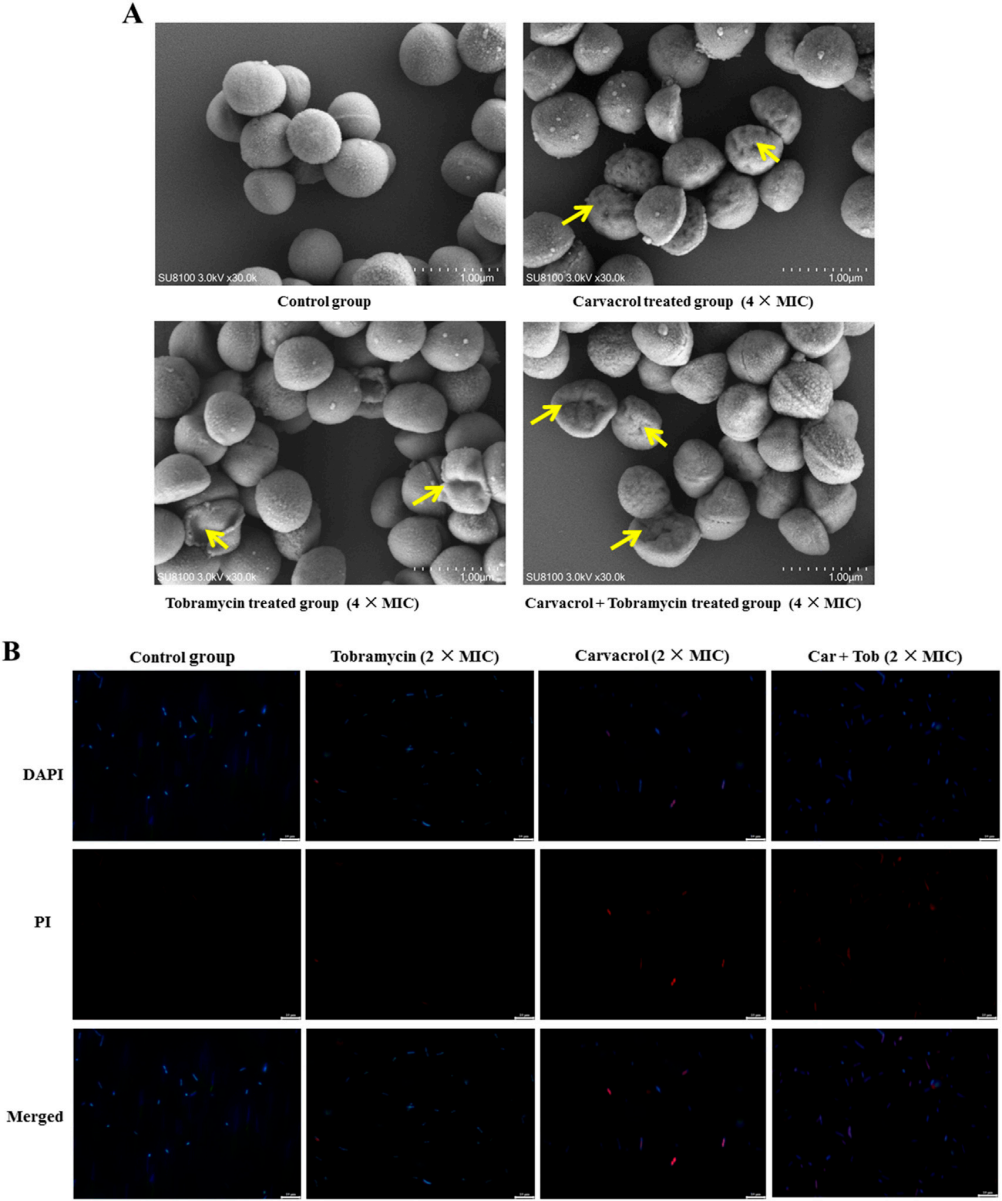
Antibacterial mechanisms of carvacrol, tobramycin and their combination against MRSA **(A)** SEM images of the MRSA membrane. Scale bar: 1.00 μm **(B)** Fluorescence micrographs of MRSA cells stained with DAPI and PI. Scale bar 10 μm.

We performed SEM assay and DAPI/PI staining to confirm whether carvacrol combining with tobramycin exert their antibacterial effects through a membrane-targeting mechanism. The changes of the cell membrane morphology of MRSA after treatment with carvacrol, tobramycin and carvacrol combining with tobramycin were directly observed by SEM (Figure 5A). Apparently, the membranes of MRSA in the control group were morphologically intact with a flat and smooth surface. In contrast, the cell surface of MRSA after carvacrol treatment was perforated with obvious holes on it, while the integrity of the membranes of MRSA was evidently wrinkled and concaved after treatment by tobramycin. However, both perforation and shrinkage of membrane were found in the group treated by carvacrol combining with tobramycin.

We further determined the integrity of bacterial membranes using fluorescent probes DAPI and PI. As shown in Figure 5B, only blue fluorescence were observed in the blank control group, indicating that the bacteria were morphologically intact and not dead. By contrast, both blue and red fluorescence were observed after MRSA treated with carvacrol, tobramycin and carvacrol combining with tobramycin, revealing that these compounds can disrupt cell membranes of MRSA and thus lead to bacterial death.

### 3.6 *In vivo* efficacy in mouse model

The above experiments have proved that carvacrol combining with tobramycin showed good antibacterial activities *in vitro*. For further evaluating its therapeutic effect *in vivo*, a murine infection model was established by intraperitoneally injection of MRSA.

**FIGURE 6:**
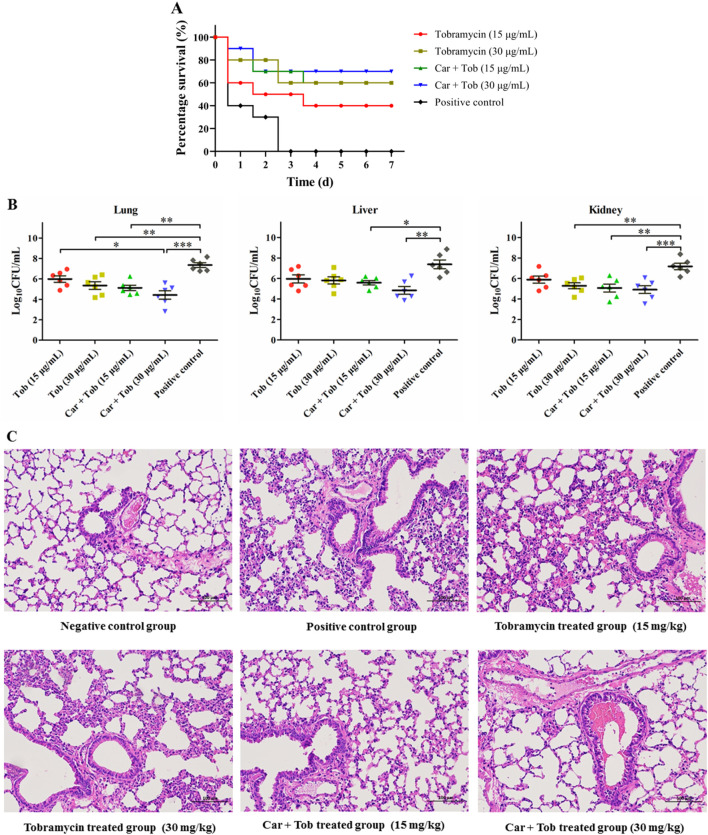
*In vivo* efficacy of tobramycin (Tob) and carvacrol combining with tobramycin (Car + Tob) **(A)** Survival curves for the infected mouse model **(B)** Bacterial loads of MRSA in lung, liver and kidney of infected mice **(C)** Lung histopathological changes of mice in normal control group, positive control group and treated groups. Values are means and the standard errors of the means. *p < 0.05, **p < 0.01 and ***p < 0.001.

All mice in the positive group died with typical clinical signs of disease within 3 days postchallenge. Treatment with 30 and 15 mg/kg of tobramycin alone, and carvacrol combining with tobramycin, respectively, displayed dose-dependent protection (Figure 6A). Analysis based on time revealed 70% survival observed in animals treated with 30 mg/kg of carvacrol combining with tobramycin.

For further *in vivo* evaluation of the synergistic effects of carvacrol, the lung, liver and kidney of the infected mice were examined for their bacterial load after treatment for 3 days. Compared with the control group, treatment with carvacrol combining with tobramycin at doses of 30 and 15 mg/kg significantly (*p* < 0.05) reduced the bacterial loads in the lungs by 2.93 and 1.36 log_10_ colony forming units (CFU), respectively. Tobramycin also exhibited significantly (p < 0.05) reductions of 2.23 and 1.99 log_10_ CFU in the lungs at doses of 30 and 15 mg/kg, respectively. Only the MRSA loads in the liver of the rats treated with carvacrol combining with tobramycin at 30 and 15 mg/kg were all significantly lower (*p* < 0.05) than that in the control group. In the kidney, two dosages of carvacrol combining with tobramycin, as well as tobramycin at doses of 30 mg/kg, significantly (*p* < 0.05) reduced the MRSA load (Figure 6B).

Base on the results that carvacrol combining with tobramycin significant reduced the bacterial load in the lungs, we further assessed whether the combining drugs attenuated the damaged tissue caused by MRSA using light microscopy method. Compared to the normal control group, the positive control group exhibited evident histopathological changes, including congestion, alveolar wall thickening, and widespread neutrophil cell infiltration within the alveolar septa and lumen. Mice treated with 15 and 30 mg/kg of carvacrol combining with tobramycin, respectively, cased attenuated histopathological changes in lung tissue when compared to the groups that treated with the same dose of tobramycin alone (Figure 6C).

## 4 Discussion

To address the great threat to public health caused by drug-resistant bacterial infections, many plant extracts have been revealed and studied. Oils extracted from Oregano are preponderant constituted by mono- and sesquiterpenes, in which carvacrol (16.75%) and thymol (21.98%) are detected as main components in our extraction processes by GC-MS analysis.

Carvacrol and thymol have both been proved to be potent against a wide range of pathogenic bacteria, including *S. aureus*, *E*. *coli*, *L. monocytogenes* (*L. monocytogenes*), *Salmonella serovar Typhimurium* (*S. typhimurium*) and *P. aeruginosa* (Kachura and Suntres, 2020; Hao et al., 2021; Oussalah et al., 2007; Chroho et al., 2024). To begin with, we compared the *in vitro* antibacterial activities of essential oil that extracted from wild and cultivated oregano with different colors of flowers, respectively, as well as their two bioactive components (carvacrol and thymol), against several standard strains. The present study revealed that the essential oil extracted from wild and cultivated oregano with white flower displayed the higher antibacterial activities than that extracted from cultivated oregano with purple flower. Although the chemical structure of carvacrol is analogous to that of thymol (Figure 1), it showed more potent antibacterial activities with MIC values range from 0.005–0.04 μg/mL for the selected strains. The clinical strains might develop certain resistances to common antibiotics after exposing for a long time (Lambert, 2000), and therefore can be used as good test objects for the evaluation of the antibacterial potency of an inhibitor in the MIC value detection. In the present study, OEOs, carvacrol and thymol against clinically isolated MRSA displayed the consistent antibacterial activity with that against standard MRSA strains.

Previous studies revealed that one of the antibacterial mechanisms of both thymol and carvacrol is potent to disrupt and disturb the bacterial cell membrane due to their hydrophobic nature (Kachura and Suntres, 2020; Gill and Holley, 2006). This may be conducive to carvacrol and thymol to overcome the problem of bacteria resistance by combination conventional antibiotics. Few studies have reported that carvacrol and thymol could significantly potentiate the antibacterial activities of some conventional antibiotics, for example, tetracycline, streptomycin, doxycycline and tilmicosin (Miladi et al., 2017; Liu et al., 2015; Kissels et al., 2017). For further expanding the data of synergistic effects, the interactions between carvacrol/thymol and common antibiotics were performed by checkerboard method (Tüzemen et al., 2024). Our results revealed that the significant reductions in MICs of tobramycin were noticed when tested in combinations with carvacrol. Cumulative exposure to large doses of tobramycin in patients is a greatest risk factor for inducing ototoxicity and nephrotoxicity (MacLeod et al., 2009). The synergistic effect of carvacrol provided the potent antibacterial effect of tobramycin at a lower dose, thus reducing dose-related toxicity during the treatment (Wang et al., 2020).

For further evaluating the synergistic effect of carvacrol, we performed the time-kill kinetics assays to evaluate the rate and degree of bacterial killing and *in vivo* efficiency evaluation using a systemic infection mice model. Results from time-kill assays revealed that carvacrol combining with tobramycin was more effective against *E. coli* and MRSA than tobramycin alone, displaying the rapid bactericidal action which will reduce the probability of bacterial resistance to antibiotics and shorten the treatment time. In the further *in vivo* antibacterial study, carvacrol combining with tobramycin displayed potent efficacy to improve the survival rate of mice, reduced the bacterial load and alleviated the pathological damage in the lungs of the infected mice. These results undoubtedly provide more complementary evidences for the observed synergy effect of carvacrol generated by our checkerboard assay.

The synergistic mechanisms of carvacrol were also investigated using SEM assay, a direct way to visualize bacterial membranes (Yang et al., 2022), and fluorescence microscopy assays which could preliminarily explore the membrane-disrupting ability of active molecules (Sun et al., 2022). A possible synergistic mechanism was proposed that the perforated bacterial surface caused by carvacrol made hydrophilic tobramycin easily pass through, which might potentiate the antibacterial effect of tobramycin by binding to bacterial ribosomes and inhibiting protein synthesis (Darby et al., 2023).

## 5 Conclusion

This study explored the antibacterial activities of OEOs, as well as carvacrol and thymol. Essential oil extracted from white flower oregano demonstrated higher *in vitro* antibacterial activities than that extracted from purple flower oregano against either standard strains or clinical isolates of MRSA. Carvacrol also was provided to be the more effective antibacterial agents than thymol. Combined sensitivity tests revealed that carvacrol displayed more synergistic effects with most common antibiotics, especially of tobramycin, than that of thymol. The synergistic effects of carvacrol were further confirmed using the time-kill assays and systemic infection mice model, in which carvacrol combining with tobramycin displayed more potent antibacterial effect than tobramycin alone. This mechanism was preliminary proposed that carvacrol could perforate bacterial surface and induced tobramycin easily pass through the lipid bilayer of bacterial membrane. These findings provide a theoretical foundation for the therapeutic applications of OEOs, carvacrol and thymol.

## Data Availability

The original contributions presented in the study are included in the article/supplementary material, further inquiries can be directed to the corresponding author/s.
