# Supplementary material for: Antibacterial activities of oregano essential oils and their active components
Source: Front Pharmacol. 2025 Apr 8;16:1579283. doi: 10.3389/fphar.2025.1579283 (PMC12011810; doi:10.3389/fphar.2025.1579283)
Supplement: Supplementary file 1 [file DataSheet1.docx]

Antibacterial activities of oregano essential oils and their active components

Lei Tao^1#^, Yan Liang^2#^, Zhi Xia^2^, Xinsheng Wang^3^, Xiaodong Wang^3^, Zhe Chao^4^, Jie Guo^1*^,

*^1^Lanzhou Vocational Technical College, Lanzhou, 730070, China;*

*^2^College of Agronomy, Henan Agricultural University, Zhengzhou, 450002, China;*

*^3^Oregano Technology Research Institute, Henan Shennong Authentic Medicinal Materials Co., Ltd, Zhengzhou, 450003, China;*

*^4^College of Veterinary Medicine, Gansu Agricultural University, Lanzhou 730070, China;*

**Corresponding author:** Tel.: +86 931 7618313; fax: +86 931 7618313. E-mail address: gjtiti@126.com (Jie Guo).

**Author:** Tel.: 86 931 7618313; E-mail address: taoleiemail2024@163.com (Lei Tao) and [Liangyanemail2024@126.com](mailto:Liangyanemail2024@126.com) (Yan Liang). Lei Tao and Yan Liang contributed equally to this work.

**Address:** Lanzhou Vocational Technical College, No. 37, Liusha road, Anning District, Lanzhou, 730070, PR China.

**Supplemental Data**

**Table of Contents**

**Table S1.** MIC values of OEOs, thymol and carvacrol against clinical mecA-positive isolates of MRSA………1

**Figure S1.** Heat plots of checkerboard assays for carvacrol/thymol in combination with different antibiotics against *E.coli* and MRSA……………………………………………………………………………………………3

**Table S1.** MIC values of OEOs, thymol and carvacrol against clinical mecA-positive isolates of MRSA.

| Stain^a^ | MIC (μg/mL) | | | | | |
| --- | --- | --- | --- | --- | --- | --- |
|  | OEO-1 | OEO-2 | OEO-3 | OEO-4 | Thymol | Carvacrol |
| SH1  SH2  SH3  SH4  SH5  QY4  SH7  ZY12  QY8  HG1  HG2  SX5  SX10  SX11  SX13  CX1  CX2  CX3  CX10  CX13  SX15  ZY6  ZY7  CX6  CX8  CX9  CX5  HG3 | 1  1  1  1  0.25  0.5  1  1  2  1  1  1  0.25  1  1  1  0.5  1  0.25  1  1  2  2  1  0.5  1  1  2 | 1  0.25  1  1  2  1  1  1  0.25  0.5  1  1  1  2  1  0.25  0.25  1  1  0.5  1  1  0.25  1  0.5  1  1  0.5 | 1  2  1  1  4  1  1  2  0.5  1  1  1  1  0.5  2  2  0.5  1  1  1  1  2  2  1  0.5  1  1  0.5 | 0.25  0.125  0.25  0.25  1  0.5  1  0.25  0.5  0.25  0.5  1  0.25  0.5  0.25  0.5  1  0.25  0.25  0.125  1  0.5  0.5  0.25  0.25  0.25  0.5  0.25 | 0.32  0.16  0.16  0.08  0.32  0.16  0.32  0.16  0.04  0.08  0.16  0.32  0.16  0.16  0.32  0.08  0.02  0.16  0.16  016  0.64  0.32  0.08  0.16  0.04  0.16  0.16  0.04 | 0.04  0.02  0.08  0.08  0.16  0.04  0.08  0.02  0.01  0.02  0.04  0.04  0.04  0.08  0.04  0.02  0.01  0.02  0.08  0.08  0.16  0.04  0.04  0.02  0.01  0.04  0.08  0.02 |

| Stain^a^ | MIC (μg/mL) | | | | | |
| --- | --- | --- | --- | --- | --- | --- |
|  | OEO-1 | OEO-2 | OEO-3 | OEO-4 | Thymol | Carvacrol |
| QY2  SH18  SH8  SH9  SH10  SH13  SH14  SH16  ZY1  ZY2  ZY8  ZY15  QY10  QY6  HG4  HG5  CX14  CX17  SH20  ZY4  SX6  ZY5  ZY14  CX11  CX19  ZY11  HG6  HG7 | 0.5  1  1  1  0.25  1  1  0.5  0.5  1  1  1  2  0.5  0.5  1  1  1  0.25  1  0.5  1  2  0.5  1  1  0.25  1 | 1  1  0.5  1  1  1  0.5  2  1  1  1  0.5  1  0.25  1  1  1  2  0.25  1  1  0.5  1  1  1  2  0.5  1 | 1  1  2  2  1  1  0.5  1  0.5  1  1  1  4  0.5  1  2  2  0.5  1  1  1  1  2  0.5  1  1  0.5  1 | 0.5  0.5  0.25  0.25  0.25  1  0.25  0.5  0.5  0.125  0.25  0.25  1  0.125  0.25  0.25  2  0.5  0.5  0.25  0.5  1  0.25  0.25  0.125  0.5  0.5  0.25 | 0.16  0.32  0.16  0.16  0.08  0.32  0.08  0.16  0.16  0.32  0.32  0.16  0.64  0.04  0.16  0.32  0.64  0.32  0.16  0.16  0.16  0.32  0.16  0.16  0.16  0.32  0.08  0.32 | 0.08  0.08  0.04  0.04  0.02  0.16  0.04  0.04  0.02  0.04  0.08  0.04  0.16  0.01  0.02  0.08  0.08  0.04  0.04  0.04  0.08  0.08  0.04  0.04  0.02  0.04  0.04  0.16 |

^a^Strains were collected from dairy farms located in 4 different provinces in China.


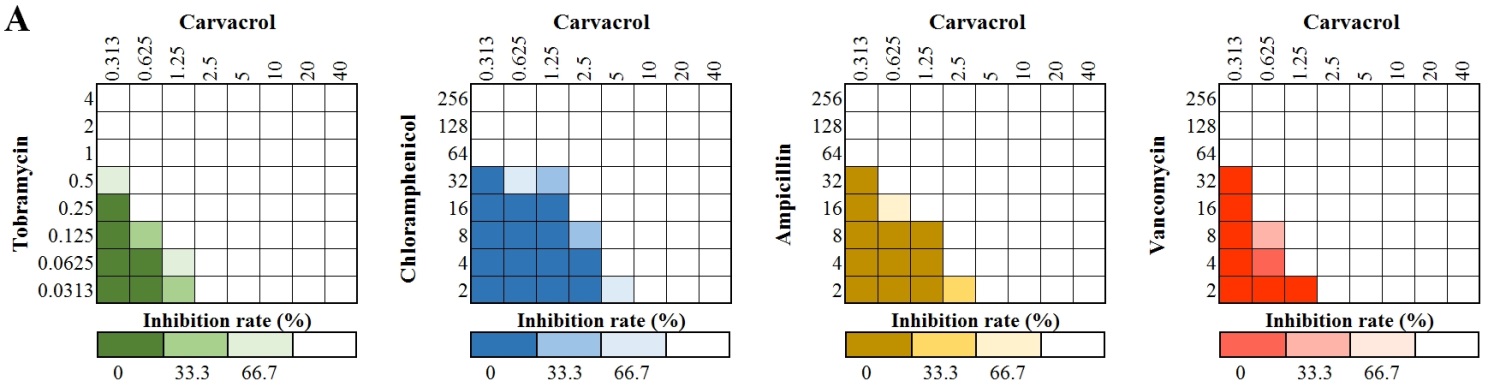


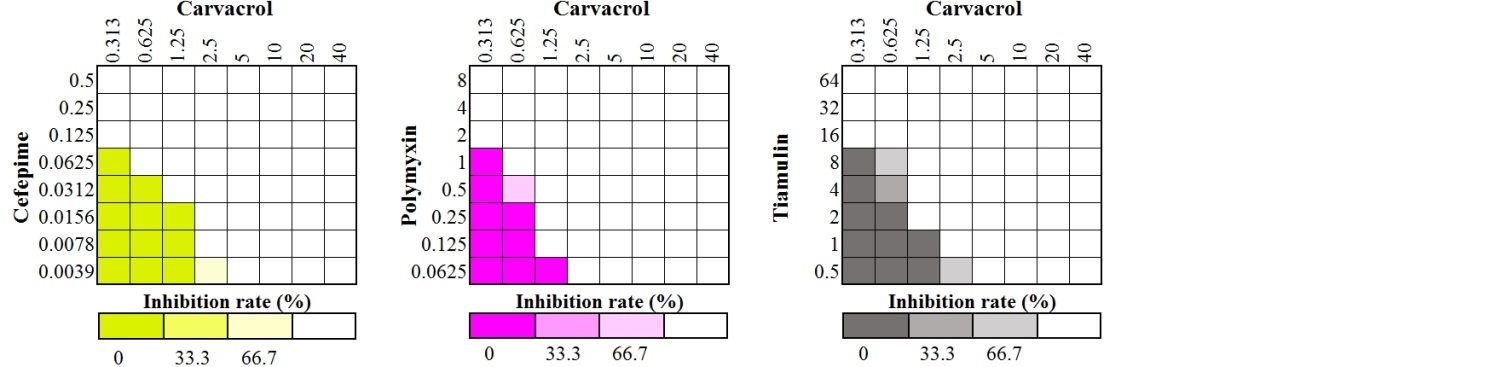


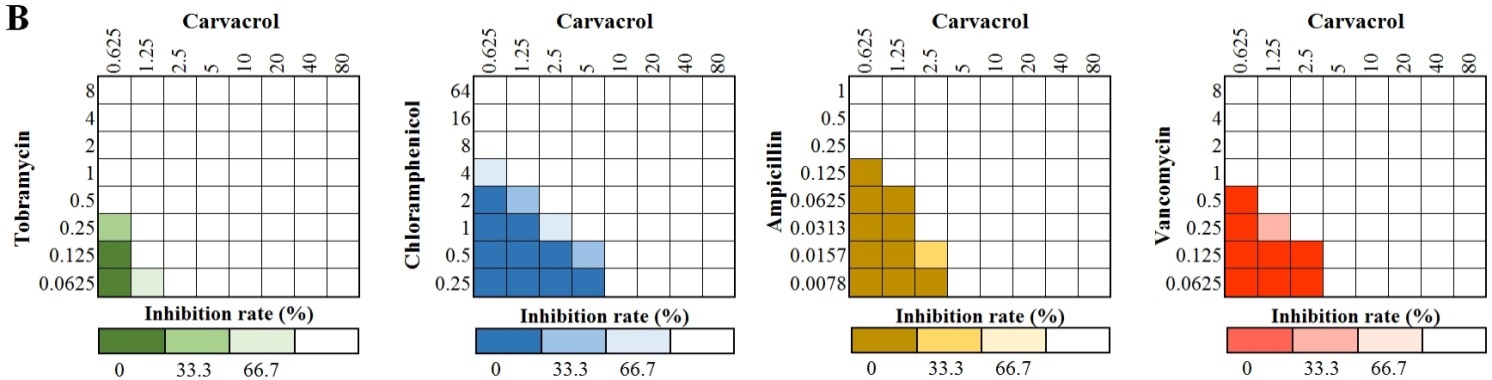


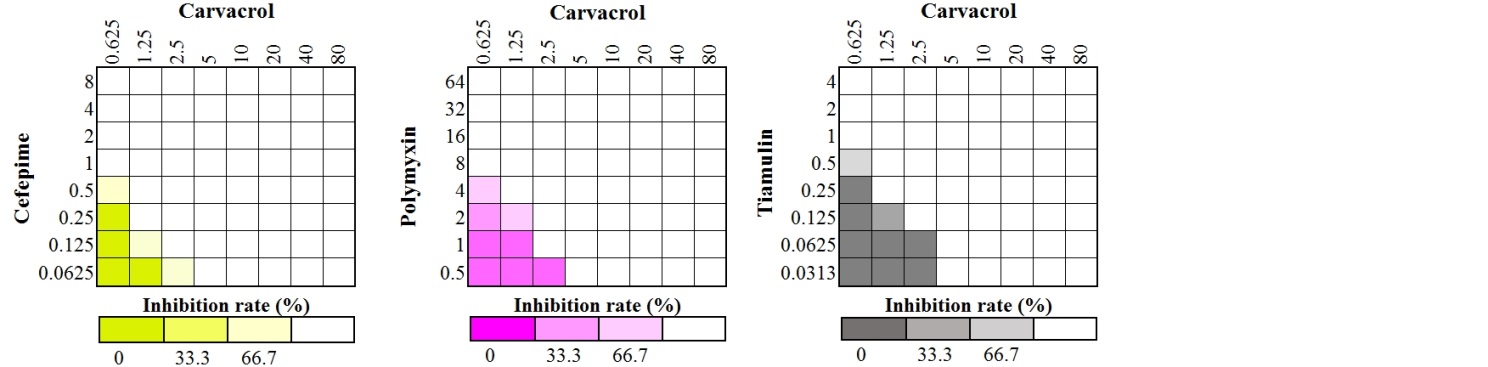


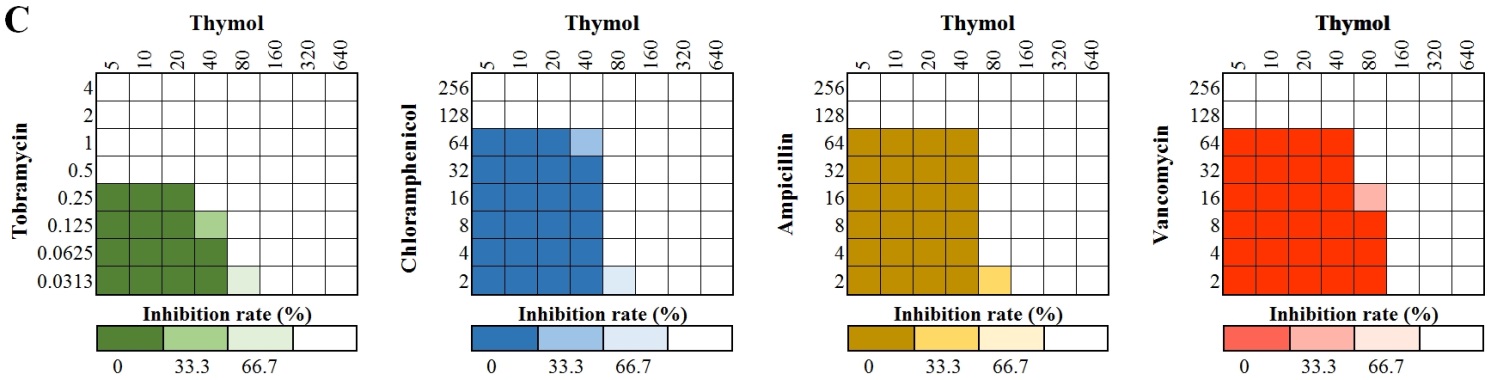


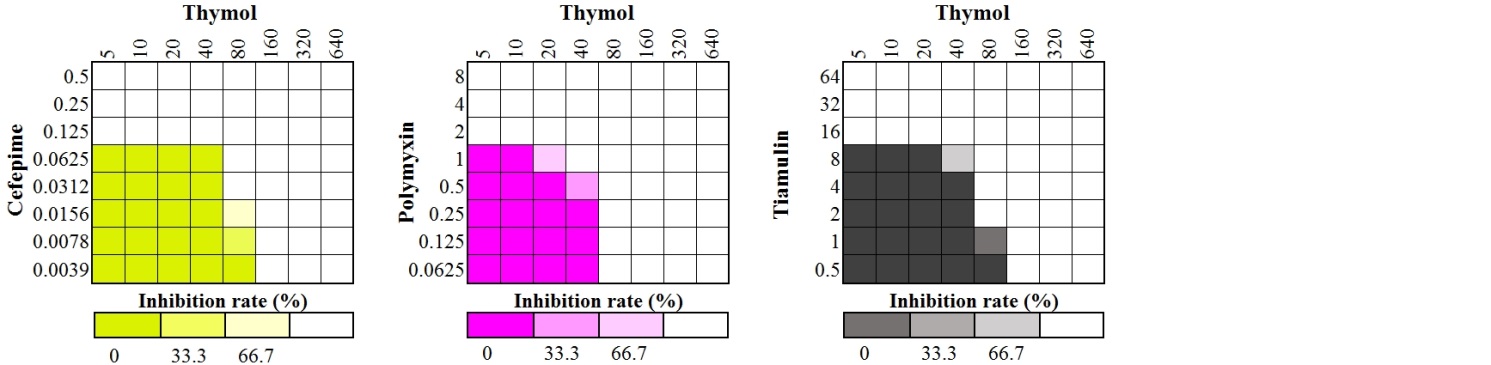


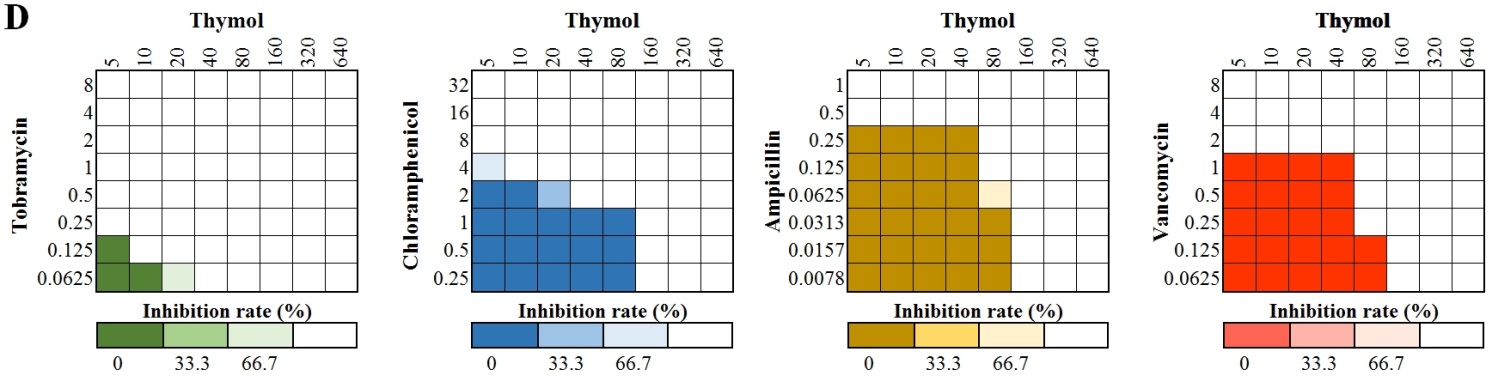


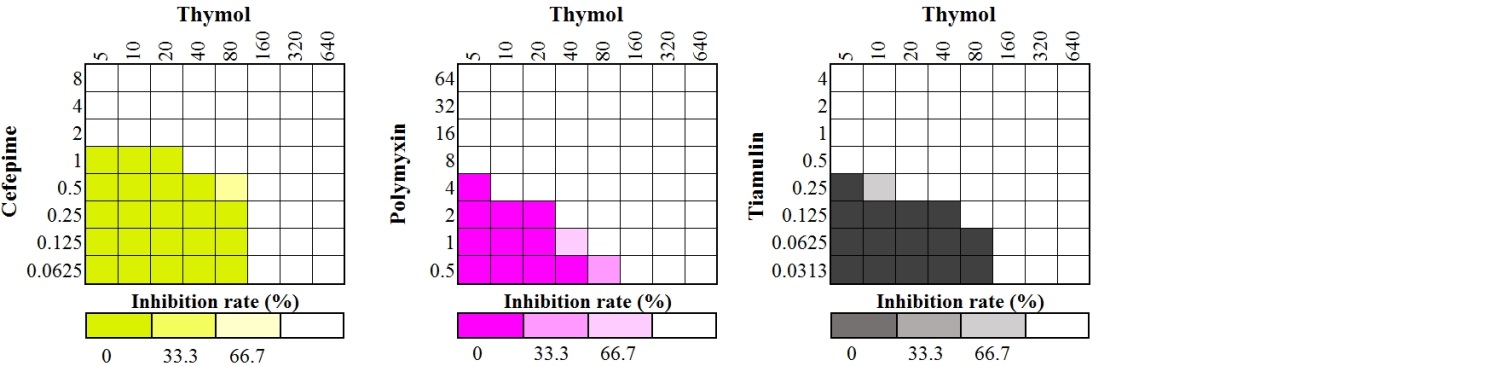


**Fig S1**. Heat plots of checkerboard assays for carvacrol/thymol in combination with different antibiotics against *E.coli* and MRSA, respectively.
